# Griffiths phase behaviour in a frustrated antiferromagnetic intermetallic compound

**DOI:** 10.1038/srep15801

**Published:** 2015-10-30

**Authors:** Krishanu Ghosh, Chandan Mazumdar, R. Ranganathan, S. Mukherjee

**Affiliations:** 1 Department of Physics, The University of Burdwan, Golapbag, Bardhaman - 713104, West Bengal, India; 2 Condensed Matter Physics Division, Saha Institute of Nuclear Physics, 1/AF, Bidhannagar, Kolkata 700064, India

## Abstract

The rare coexistence of a Griffiths phase (GP) and a geometrically frustrated antiferromagnetism in the non-stoichiometric intermetallic compound GdFe_0.17_Sn_2_ (the paramagnetic Weiss temperature *θ*_p_ ~ −59 K) is reported in this work. The compound forms in the *Cmcm* space group with large structural anisotropy (*b*/*c* ~ 4). Interestingly, all the atoms in the unit cell possess the same point group symmetry (Wycoff position 4*c*), which is rather rare. The frustration parameter, f = |*θ*_p_|/T_N_ has been established as 3.6, with the N*é*el temperature T_N_ and Griffiths temperature T_G_ being 16.5 and 32 K, respectively. The T_G_ has been determined from the heat capacity measurement and also from the magnetocaloric effect (MCE). It is also shown that substantial difference in GP region may exist between zero field and field cooled measurements - a fact hitherto not emphasized so far.

In magnetic systems, it was shown that the observation of a Griffiths phase (GP)^1^ may have many different origins, *e.g*., phase separation, occurrence of clusters of sizes ranging from nanometers to micrometers^2^, competing intra- and interlayer magnetic interaction, microtwining, *etc*.^3^,^4^,^5^. Experimentally, most of the GP compounds are known to exhibit bulk ferromagnetic (FM) order. In some of the antiferromagnetic (AFM) compounds which show characteristic reminiscent of a GP^6^,^7^, the paramagnetic Weiss temperature (*θ*_p_) is found to be positive, suggesting the presence of strong FM interactions. The only GP compound known to us, that exhibits negative *θ*_p_, is an oxide compound Ca_3_CoMnO_6_^8^. This implies that in most of the GP compounds the FM interactions compete quite strongly with dominant AFM interaction, resulting in positive *θ*_p_. In order to realize the GP in an AFM system with |*θ*_p_|/T_N_ < 1, one needs to search for a system that has not only the favourable types of structural defects, but also should have the magnetic interaction of A-type 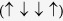 or C-type 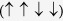, where 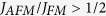, *J* being the exchange interaction^8^. Thus an appropriate choice of AFM compound that has suitable structural defects and the right kind of competing magnetic interaction, may lead us to a GP even in a geometrically frustrated magnetic (GFM) system. This work focuses on such problem, namely, to search for a geometrical frustrated antiferromagnet with a GP. This is yet to be realized in view of complex magnetic interactions in AFM intermetallic compounds.

It was earlier found that most of the *RETM*_*x*_Sn_2_ (*RE* = rare earths, *TM* = transition metals; *x* < 1) compounds form in a defect orthorhombic CeNiSi_2_-type crystal structure (space group: *Cmcm*)^9^ which is a filled variant of ZrSi_2_-type structure (space group: *Cmcm*). In both structures, all the atoms occupy distinct 4*c* Wyckoff positions (0, *y*, 0.25)^9^, and do not show any inter-element cross-substitutional effects^10^. While the CeNiSi_2_-type structure may be considered as an intergrowth of binary AlB_2_ and ternary BaAl_4_-type slabs, the ZrSi_2_-type structure may be viewed as an intergrowth of binary AlB_2_ and binary CaF_2_-type slabs. If all the transition metal atoms are removed from the ternary BaAl_4_-type segment it can be considered as a CaF_2_-type structure [see Fig. 1(a) of ref. 11]. The binary *RE*Sn_2_ compounds form in the ZrSi_2_-type crystal structure^12^,^13^,^14^,^15^. In the complex solid solutions of *RETM*_*x*_Sn_2_ (*x* < 1) compounds, the transition metals are randomly distributed leaving many *TM* sites vacant^12^,^16^. This, in turn, results in the local variation of *RE* - *RE* bond length^11^. The random disruption of magnetic exchange interaction caused by the varying bong lengths affects the long range magnetic order throughout the sample, but are expected to be found in many microscopically small regions inside the samples. According to the Griffiths model, the disorder driven random distribution of magnetic interactions result in different sets of exchange constants for different lattice points throughout the material^1^,^17^. *RETM*_*x*_Sn_2_ (*x* < 1) compounds that have the similar characteristics, therefore appear to be potential candidates for observing a GP. In the case of *RE*Fe_*x*_Sn_2_ series (*RE* = Tb - Tm), it was shown earlier through neutron diffraction experiments that in addition to A- or C-type AFM ordering (favourable to a GP in AFM systems), the systems also have a sizeable number of frustrated rare earth ions, that ensures 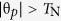
18. In our quest to search for GP in a GFM intermetallic system, we have synthesized and studied the magnetic properties of GdFe_0.17_Sn_2_, a material that satisfies most of the above mentioned criteria. The Gd-based system (L = 0) was chosen due to its negligible magnetic anisotropy resulting from the higher order exchange interaction^19^.

## Results and Discussion

**Figure 1:**
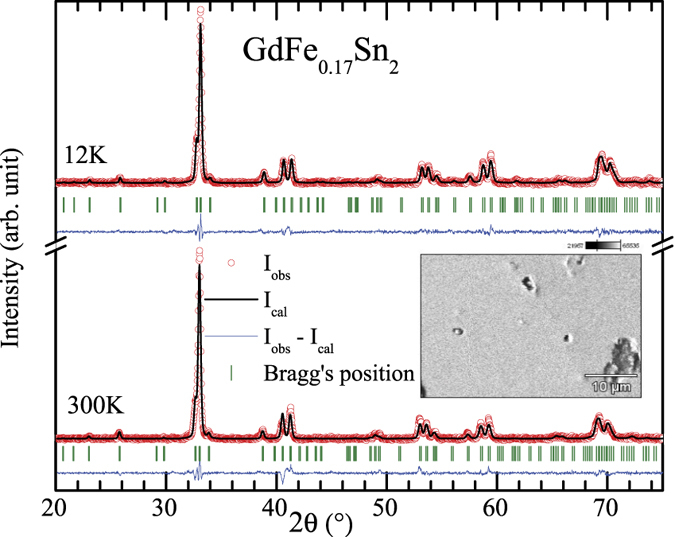
(Bottom) XRD pattern of GdFe_0.17_Sn_2_ measured at room temperature. The inset shows SEM image. (Top) XRD pattern of GdFe_0.17_Sn_2_ measured at 12 K.

**Figure 2:**
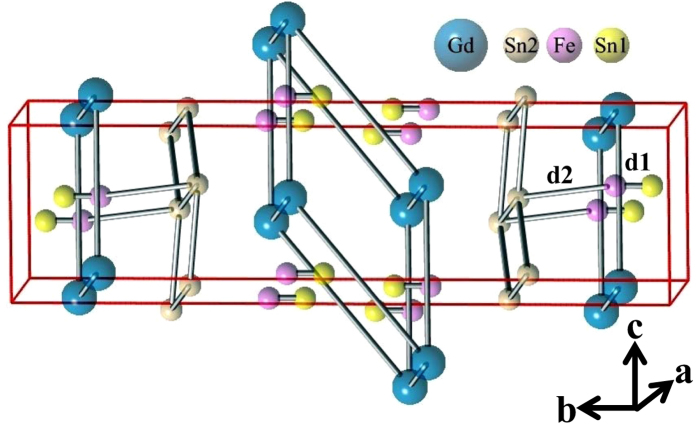
Crystallographic structure of GdFe_0.17_Sn_2_.

The powder x-ray diffraction (XRD) patterns taken at room temperature of GdFe_0.17_Sn_2_ were analyzed by considering that the material has a CeNiSi_2_-type orthorhombic structure (space group: *Cmcm*). All the peaks in the XRD pattern could be indexed using this space group (Fig. 1, bottom). The lattice parameters, *a* = 4.443(1)Å, *b* = 16.43(1)Å and *c* = 4.371(1)Å are close to that reported earlier for GdFe_0.17(2)_Sn_2_^16^. However, the full Rietveld analysis, allowing the variation of occupancy factor of Fe and Sn atoms, suggests the actual composition to be GdFe_0.19_Sn_1.93_. We find the average interatomic distances Fe-Sn1 (d1) as 0.9 Å, Fe-Sn2 (d2) as 3.1 Å and the angle Sn1-Fe-Sn2 as 135.2° (Fig. 2). In isostructural stoichiometric CeNiSi_2_, we find that the Ni-Si1 and Ni-Si2, distances are nearly identical, close to 2.31 Å. The Sn1-Fe-Sn2 angle in GdFe_0.17_Sn_2_ is also found to be quite stretched in comparison to that found in CeNiSi_2_, where the Si1-Ni-Si2 angle is close to 117.7°^20^. Shorter average interatomic distances (Fe-Sn1) generally reflect the presence of vacancies (Fe and/or Sn) in the crystal structure, and earlier reported in quite a few *RETM*_*x*_Sn_2_ series of compounds (*TM*-Sn ~ 2–2.5 Å)^12^,^21^. The scanning electron microscope (SEM) picture (Fig. 1; (bottom: inset)) and energy dispersive analysis of X-ray (EDAX) suggest an essentially single phase nature with average composition as GdFe_0.19_Sn_1.91_ which is close to the value obtained through Rietveld analysis. The diffraction patterns taken at lower temperatures do not suggest any major structural phase transformation (Fig. 1; top).

**Figure 3:**
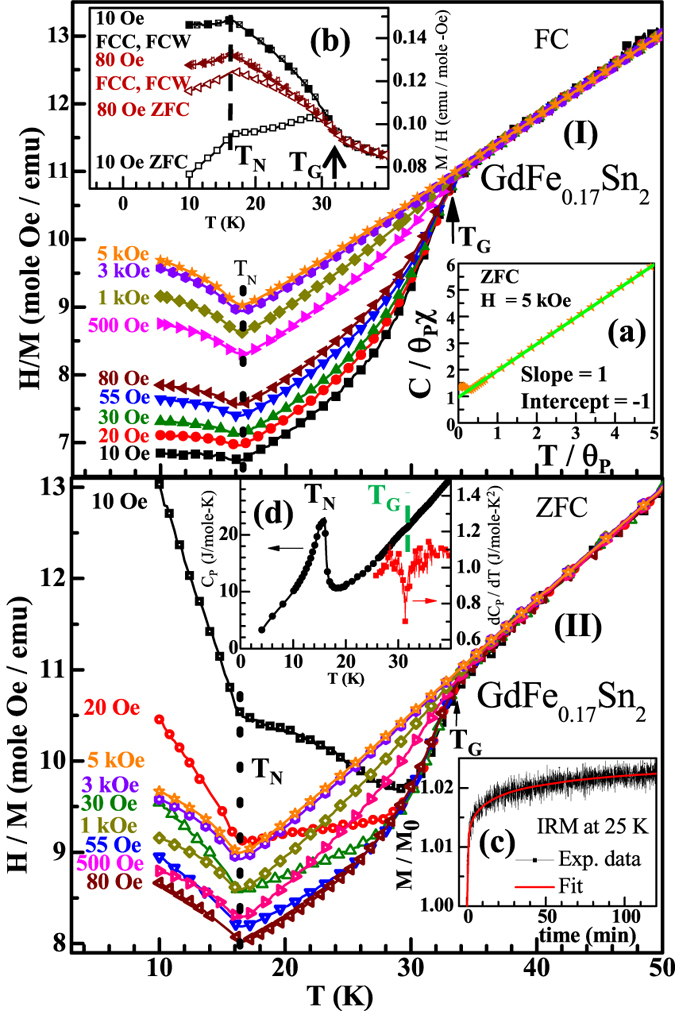
Temperature dependence of the inverse magnetic susceptibilities of GdFe_0.17_Sn_2_ measured at different externally applied magnetic field under FC (top-I) and ZFC (bottom-II) configuration during warming cycle; (Inset (**a**)): Normalized inverse susceptibility versus temperature at H = 5 kOe. C is the Curie constant and *θ*_p_ is the paramagnetic Weiss temperature; (Inset (**b**)): Magnetic susceptibilities of the same sample measured under ZFC, FCC and FCW conditions in fields of 10 and 80 Oe: (Inset (**c**)): Normalized magnetic relaxation data along with the fit of stretched exponential are presented. (Inset (**d**)): Temperature dependence of heat capacity in absence of any magnetic field is shown using the left side axis. The anomaly observed due to GP transition can be observed more clearly by taking derivative of the heat capacity around T_G_ as shown using the right hand axis of the inset.

**Figure 4:**
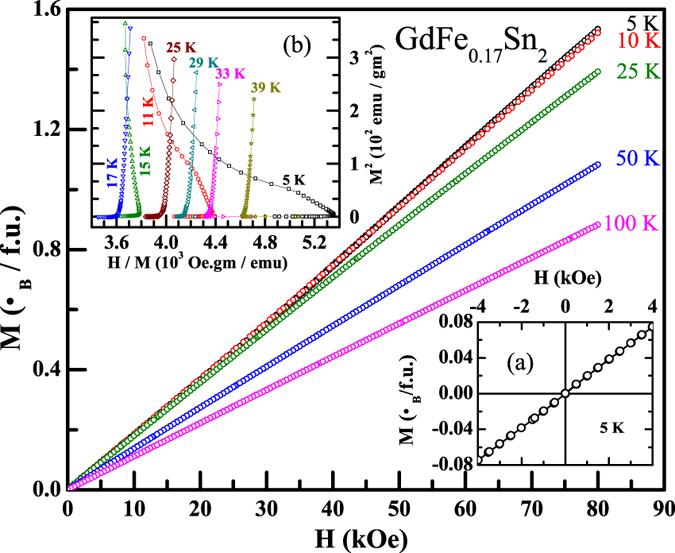
Isothermal magnetization at different temperatures of GdFe_0.17_Sn_2_ are presented for 0 ≤ H ≤ 80 kOe. (Inset (**a**)): All the isothermal magnetization measurements were performed up to a range of ±80 kOe. Here, only the data in only the range of −5 ≤ H ≤ 5 kOe measured at 5 K are shown. (Inset (**b**)): Arrott plot in the temperature range 5–39 K.

The magnetic susceptibility (*χ*) measurements (2–300 K) under the influence of external magnetic field, in excess of 5 kOe, suggest that the compound orders magnetically at ~16.5 K (Fig. 3). The negative value of *θ*_p_ (−59 K), estimated from inverse magnetic susceptibility in the paramagnetic region and the absence of spontaneous magnetization in the Arrott plot (M^2^ vs. H/M)^22^ (Fig. 4; inset (b)), suggest that GdFe_0.17_Sn_2_ orders antiferromagnetically at T_N_ ~ 16.5 K. The isothermal magnetizations measured below T_N_, do not exhibit any hysteresis behaviour and have a near linear magnetic field dependence, as expected for an AFM system (Fig. 4) (Fig. 4; inset (a)).

The estimated value of frustration parameter, *f* = |*θ*_p_|/T_N_ = 3.6, suggests that this compound is a frustrated magnetic system (Fig. 3; inset(a)), as per the criterion suggested by A. P. Ramirez^23^. This magnetic frustration has its origin in the crystal structure itself. In the CeNiSi_2_-type crystal structure, the rare earth ions are arranged as infinite sheets of face-sharing *RE*_6_ trigonal prisms, and also as *RE*_4_ tetrahedrons stretched along [010]^18^. The neutron diffraction measurements on *RE*Fe_*x*_Sn_2_ (*RE* = Tb - Tm, 0.1 < *x* < 0.15) had earlier established an A- (or C-) type AFM structure where some of the rare earth ions occupying the position of the trigonal faces of the prism and/or in the tetrahedron exhibiting frustration of the magnetic moments [see Fig. 4(b), 7(b), 11(b) of ref. 18]. This is in agreement with the fact that these compounds also have frustration parameter *f* ~ 2–3^18^, similar to that observed in GdFe_0.17_Sn_2_. The origin of magnetic frustration in *RE*Fe_*x*_Sn_2_ (*RE* = Tb - Tm, 0.1 < *x* < 0.15) primarily depends on the crystal structure. Therefore GdFe_0.17_Sn_2_, being in the same crystallographic structure, is also expected to possess geometrically frustrated magnetic moments. The magnetic structure of GdFe_0.17_Sn_2_, however, has not be directly checked using neutron diffraction technique due to the large cross-section of neutron absorption of Gd.

The value of effective magnetic moment per formula unit (*μ*_*eff*_) calculated from the inverse magnetic susceptibility in the paramagnetic range found to be 8.19*μ*_*B*_ which is slightly higher than that of free Gd^3+^ -ions (7.94*μ*_*B*_). One may attribute the origin of larger moment as due to the Fe atoms present in this compound. Here, it may be noted that the values of *μ*_*eff*_ for all other members of the series *RE*Fe_*x*_Sn_2_ (*RE* = Tb – Tm, 0.1 < *x* < 0.15) are also reported to be higher than that of their corresponding free ion values, where neutron diffraction measurements failed to detect any ordered moment of Fe^18^. Additionally, GdSn_2_ that forms in the same space group (*Cmcm*), also exhibits *μ*_*eff*_ of similar magnitude (8.16*μ*_*B*_)^24^. Therefore, in our opinion, the slightly larger value in *μ*_*eff*_ observed in our compound might originate from the positive polarization of conduction electrons^24^, or as A. P. Ramirez suggested, due to the reduction of moment density (resulting in an increase of effective magnetic moment) generally found in frustrated magnetic systems^23^.

The susceptibility (*χ*), however, measured at a field lower than 5 kOe, both in zero field cooled (ZFC) and field cooled (FC) configuration suggests the presence of another anomaly around 32 K (Fig. 3; inset (b)). The field cooled magnetic susceptibilities do not show any remanence while measuring during cooling (FCC) and subsequent warming (FCW) (Fig. 3; inset (b)). We first discuss the magnetic susceptibility measurements in FC configuration. The ZFC measurement and presence of thermoremanent behaviour in low field will be discussed later.

The inverse FC magnetic susceptibility (H < 5 kOe), in the paramagnetic region (T > T_N_) shows a downward deviation from linearity below a temperature, T_G_ ~ 32 K (Fig. 3; I). As the applied external magnetic field increases gradually from 10 Oe onwards, the extent of deviation in magnetic susceptibility systematically diminishes until a Curie-Weiss (CW) behaviour is observed down to T_N_, for a field in excess of 5 kOe (Fig. 3; I). The deviation from CW behaviour at a low measuring field, while approaching the ordering temperature (T_C_ or T_N_) from above (at T_G_), may be attributed due to the presence of small clusters in addition to the paramagnetic matrix. The downward deviation in χ^−1^(T) results from an enhancement in χ due to the contribution from the FM clusters. The deviation is suppressed in large magnetic field due to the polarization of spins outside the clusters. To establish the presence of magnetic clusters in GdFe_0.17_Sn_2_, we have also performed spin relaxation measurements by studying the Isothermal Remanent Magnetization (IRM) in the GP region. The magnetization exhibits a relaxation behaviour, that can only be fitted well using a stretched exponential form, 
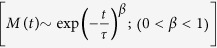
 below T_G_, with *β* ~ 0.45 (Fig. 3; inset (c)), often observed in the systems containing magnetic clusters^25^,^26^. This FM-like anomaly at 32 K cannot be attributed to the structurally related compound GdSn_2_, even if it is present in our compound below the resolution limit of XRD, as it orders antiferromagnetically at 27 K^24^. It may be noted here, in the case of ThFe_0.2_Sn_2_, the cross-substitution effect of different constituent elements had been categorically ruled out through the ^57^Fe and ^119^Sn Mössbauer spectroscopy measurements^10^. It is therefore quite unlikely that any such cross-substitution would occur in the isostructural GdFe_0.17_Sn_2_ system as well. Thus, the anomaly at 32 K appear to be an inherent characteristic of GdFe_0.17_Sn_2_.

The phenomena observed in the magnetic susceptibility of GdFe_0.17_Sn_2_ can be explained using a model proposed by Griffiths^1^. In the GP model, the long-range ordering temperature, T_C_(*x*), of a randomly diluted ferromagnet will be lower than the same of the undiluted one 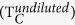. The thermodynamic properties (*e.g.* magnetization) will be non-analytical in this region 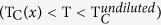 due to the formation of a low density clusters with short-range ordering. Here 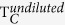 is the temperature at which this GP forms and is popularly known as the Griffiths temperature (T_G_). The temperature range between T_C_ and T_G_ corresponds to a GP, and this GP is different from the paramagnetic as well as the long-range FM phase. Though the typical behaviour of a GP are already reported in several compounds^5^,^8^,^17^,^27^, the direct confirmation of GP in any system is really a difficult task^17^.

One indirect way to confirm whether the short-range FM correlations observed in the PM state can be ascribed to the GP is the deviation of magnetic susceptibility for T << T_G_. χ^−1^ in such case should generally follow power law behaviour describing the Griffith singularity^28^


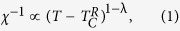


where λ is the magnetic susceptibility exponent, and 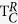 is the critical temperature of random ferromagnetic clusters where susceptibility tend to diverge. In most of the GP compounds reported so far, the magnetic transition temperature (T_G_ or T_N_) and 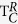 found to be very close^5^,^17^,^29^,^30^. However, for an AFM system, where *θ*_p_ < T_N_, one is constrained to test the validity of the power law behaviour close to T_N_ only, instead of a region close to 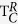. However, in cases where 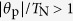 and T_N_ ~ T_G_, *i.e.*, 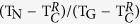 is quite high (>0.5), the temperature region available for testing the applicability of this power law turns out to be closer to T_G_, than to 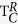, and in such cases, the above mentioned power law cannot be applied. The large value of (T_N_ - 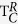)/(T_G_ - 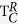) ~ [16.5 − (−59)]/[32 − (−59)] = 0.83, estimated in the case of GdFe_0.17_Sn_2_, restricts the applicability of the above mentioned power law behaviour in the present case. Although we also observed that the behaviour of χ^−1^(T) in the temperature range, T_N_ < T < T_G_, is similar to GP, or more accurately, a system having FM clusters in a temperature range above the long range magnetic ordering temperature. It may be noted here that the only other oxide GFM that also exhibits GP behaviour, Ca_3_CoMnO_6_, has similar values of *f* (~3.8), *θ*_p_ (−50 K) and T_N_ (~13 K), but the much larger value of T_G_ (~125 K) ensures that eq. (1) can be applicable close to T_N_ in that compound [(T_N_ - 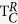) /(T_G_ - 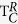) ~ 0.36]^8^, unlike in the present case of GdFe_0.17_Sn_2_.

In order to demonstrate the existence of a GP in our system, we should look into the possible reason behind the formation of such clusters. In GdFe_0.17_Sn_2_, only 17% of the transition metal-sites are randomly occupied with Fe atoms and the rest are vacant. This creates a local disorder in the crystal structure. As mentioned earlier, the presence of these vacancies are also reflected in the estimated shortened average bond lengths^12^,^21^. The random presence of vacancies in the transition metal site decreases the Gd^3+^–Gd^3+^ interionic distance locally^11^ and thereby introduces a random spatial variation of the exchange interaction (J) in the bonds between Gd^3+^ ions on a regular lattice^31^. This results in the coexistence of two phases with different J values within the same crystalline phase. In the first phase, the Fe-sites are vacant and this forms the major phase, while the minor phase, containing Fe atoms at the transition metal-sites, are randomly distributed within the major phase^12^. Similar coexistence of magnetic phases has already been observed in isostructural CeNi_0.84_Sn_2_^32^. In the original work of Griffiths, it was shown that, if a great enough fraction, *x* > *x*_*c*_, of the bonds of a ferromagnet have J = 0, then a GP will form in the temperature range, T_C_ < T < T_G_, with non-analytic free energy in an external magnetic field. The observation of a GP is possible in a system where the random disorder results in random spatial variation of J. The effect of disorder is to partition the pure system into small FM clusters. The disorder also needs to be quenched^25^. The disorder is quenched in our system also, since the Fe atoms occupying the transition metal sites are fixed in the lattice. In GdFe_0.17_Sn_2_, it will be therefore justified to associate the major phase having vacant Fe-sites with long range AFM ordered state. The minor phase results from the random dilution of the AFM exchange interaction and is distributed in isolated small-sized FM clusters having positive J value. It may be pertinent to note here that in case of perovskite ruthenates, the variation of bond angle are argued to be responsible for observation of a GP^33^. Thus, intermetallic GdFe_0.17_Sn_2_ appear to be one of the rare breed of compounds, apart from the oxide Ca_3_CoMnO_6_, to exhibit GP characteristics in an otherwise GFM material.

In order to study the change in magnetic entropy, if any, due to the FM ordering of such a small volume fraction of material, we have also made a careful study of the heat capacity as well as the magnetocaloric effect (MCE) close to T_G_. The heat capacity of GdFe_0.17_Sn_2_ shows a large peak around 15.9 K, close to T_N_ (Fig. 3; inset (d)). The magnetic contribution to entropy at T_N_, estimated from the heat capacity data, is found to be only ~1.7*R*, instead of the theoretically expected value of 2.09*R* (*R* = universal gas constant). The reduced value of magnetic entropy suggests that nearly 20% of Gd-ions do not take part in long range ordering. Surprisingly, we find that the magnetic entropy associated with FM clusters in the GP, however insignificant, also leaves its imprint in heat capacity results, exhibiting a discernible anomaly close to T_G_ ~ 32 K (Fig. 3; inset (d), right axis).

**Figure 5:**
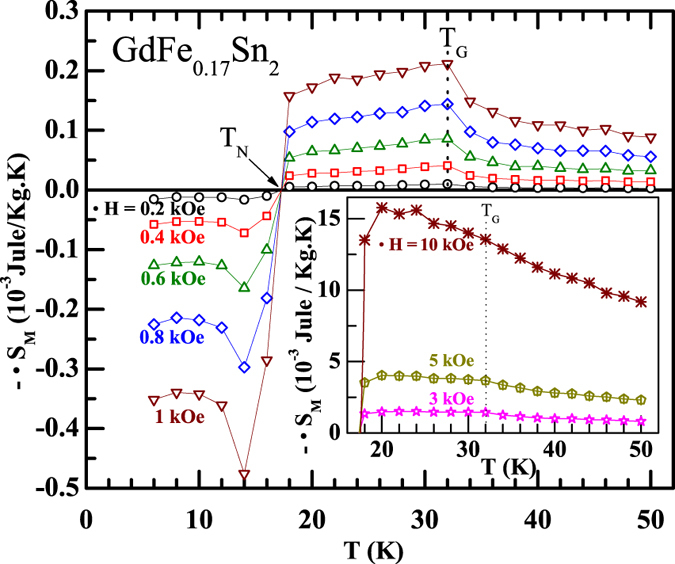
Magnetic entropy, estimated from low field isothermal magnetization measurements. (Inset): The magnetic entropy measured at higher magnetic fields.

The presence of FM-like spins within the clusters can also be confirmed through low field MCE measurements, that tracks the associated entropy change due to GP ordering. Although the volume fraction responsible for the GP is small, nevertheless the isothermal magnetic entropy (ΔS_M_) measured at low field, unambiguously show a reduction in value below T_G_ (Fig. 5). The values of ΔS_M_ have been calculated from magnetization isotherms using the Maxwell thermodynamic relation,


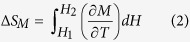


Close to T_G_, ΔS_M_(T) exhibits an well-defined peak, often seen in materials with ferromagnetic type ordering. Similar to the reduction in excess magnetization below T_G_ with the increase of magnetic field, the peak in MCE also gets suppressed for a magnetic field larger than 5 kOe (Fig. 5; inset). To the best of our knowledge, probing of a GP through MCE has not been reported earlier in literature.

We now focus on the ZFC magnetization measurement, which particularly at very low field, exhibit a markedly different nature to that which was commonly observed in FC measurements. While the FC magnetization tends to saturate at lower temperature (T_N_ < T < T_G_), the low field ZFC measurements yield very small moment values, that rise with increasing temperature until reaching some maximum value below T_G_, and then follow the FC susceptibility for higher temperatures (inverse susceptibility as shown in Fig. 3; II). As the applied magnetic field strength increases gradually, the thermoremanence decreases, and for magnetic fields higher than 500 Oe, the thermoremanence becomes barely discernible. Such thermoremanence behaviour are generally reported in spin-glass type compounds due to the presence of metastable states^34^ or in anisotropic ferromagnets^35^ due to the competing interactions of magnetic coupling energy and anisotropy energy. Although a few GP compounds, *e.g.*, Gd_5_Ge_4_, have also been reported to exhibit thermoremanent behaviour^5^, such a strikingly different behaviour of ZFC and FC magnetization has not been reported earlier for any GP compound. Our result thus shows that the typical signature of GP, generally observed through FC measurements, may yield different character when measured in ZFC configuration.

In conclusion, we have shown that the intermetallic compound GdFe_0.17_Sn_2_ can be considered as a unique system that orders antiferromagnetically with geometric frustration (*f* = |*θ*_p_|/T_N_ = 3.6), and exhibits features similar to that observed in a GP. Only one oxide compound, Ca_3_CoMnO_6_, has been reported earlier to have similar features^8^. We have found that the variation of magnetic susceptibility with field in the GP region may depend considerably on the measurement protocol, *i.e.*, ZFC and FC measurements, due to the metastable states of the spins involved in the GP. Such magnetic thermoremanent behaviour had not been reported earlier in any GP compounds. We have also shown that beside the magnetic susceptibility, GP ordering temperature can also be probed through both the MCE as well as the heat capacity measurements.

## Methods

A number of polycrystalline compounds having nominal composition GdFe_0.17_Sn_2_ were melted in a water cooled arc furnace in the flowing argon atmosphere. The samples were melted several times to ensure homogeneity. The resultant ingots were then wrapped in Ta-foil and annealed under vacuum at 800 °C for 15 days. Powder x-ray diffraction (XRD) measurements were performed in the temperature range of 12–300 K using a 18 kW rotating anode diffractometer (Model: TTRAX-III, M/s Rigaku Corp., Japan). The single phase nature as well as lattice parameters were ensured through the Rietveld refinement analysis using FULLPROF software^36^. Magnetic [M (T, H, time)] and heat capacity (in the absence of external magnetic field) measurements were performed in the temperature range 2–300 K using a commercial SQUID-VSM and PPMS Evercool-II (M/s Quantum Design Inc., USA). The Scanning Electron Microscope (SEM) (S-3400N: M/s Hitachi, Japan) and energy dispersive analysis of X-ray (EDAX) (M/s Thermo Electron Corp. USA) measurements were also performed to check the spatial homogeneity and elemental composition of the system. The Isothermal Remanent Magnetization (IRM) measurements were carried out using the following protocol: the sample was cooled in the absence of magnetic field (H = 0) from room temperature to a temperature (25 K) below T_G_. Then, with a waiting time t_*w*_ = 0 sec, the magnetic field was switched on and allowed to reach a value of 50 kOe. Once the magnetic field reaches 50 kOe, the field was switched off after t_*w*_ = 0 sec. As soon as the magnetic field diminishes to 0, the time dependence magnetization measurement was carried out, again after t_*w*_ = 0 sec.

## Additional Information

**How to cite this article**: Ghosh, K. *et al.* Griffiths phase behaviour in a frustrated antiferromagnetic intermetallic compound. *Sci. Rep.* **5**, 15801; doi: 10.1038/srep15801 (2015).
