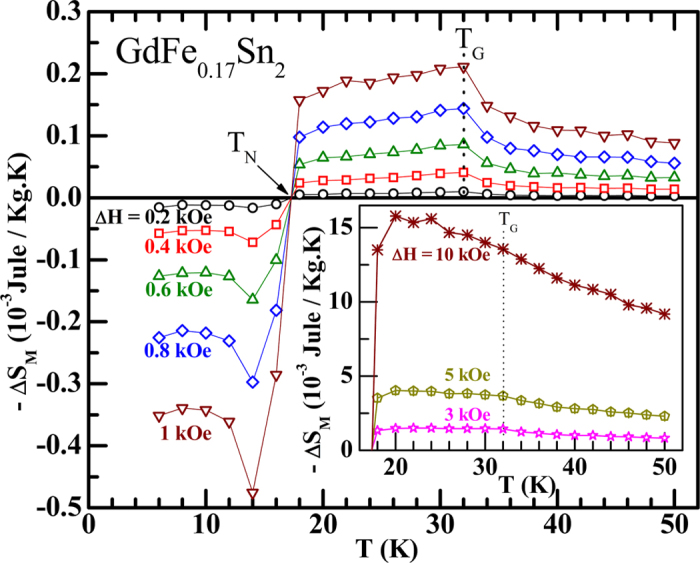# Erratum: Griffiths phase behaviour in a frustrated antiferromagnetic intermetallic compound

**DOI:** 10.1038/srep20468

**Published:** 2016-02-02

**Authors:** Krishanu Ghosh, Chandan Mazumdar, R. Ranganathan, S. Mukherjee

Scientific Reports 5: Article number: 1580110.1038/srep15801; published online: 10302015; updated: 02022016

This Article contains errors in Figure 4 and Figure 5.

In Figure 4, the y-axis ‘M (μ_B_/f.u.)’ was incorrectly given as ‘M(·_B_/f.u.)’. In Figure 5, the y-axis ‘−ΔS_M_ (10^−3^Jule/Kg.K)’ was incorrectly given as ‘−·S_M_ (10^−3^Jule/Kg.K)’. The correct Figure 4 and Figure 5 appear below as Figures 1 and 2 respectively.

## Figures and Tables

**Figure 1:**
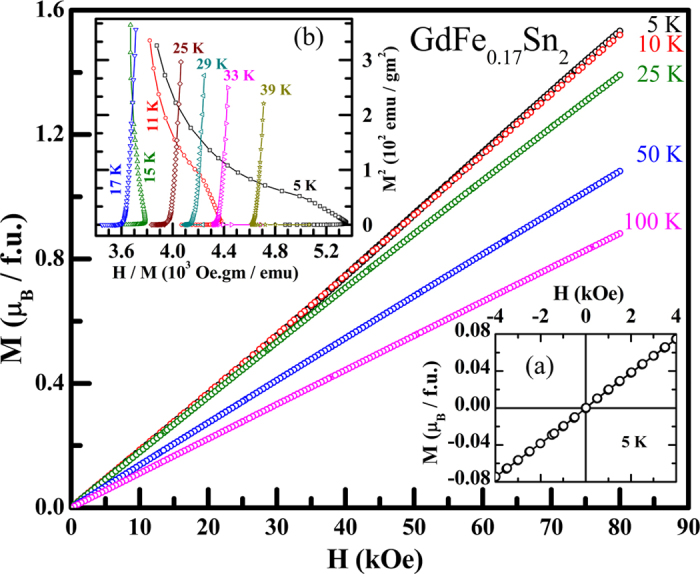


**Figure 2:**